# Editorial

**Published:** 1875-09

**Authors:** 


### Editorial.

Two journals, possessing, in a high degree, respectability and influence, have been published in this city for a number of years, under the auspices of the two Medical Colleges located here.

To a certain extent they were regarded as the organs of these institutions, and although liberal and high-toned in their character, it was difficult for the profession to consider them in any other light than partisan.

About a year since, a few medical gentlemen conceived the idea of starting another journal, which should have no bias toward any institution or party ; one in which all members of the profession could unite their energies for the advancement of medical science.

With this view, the enterprise which finally led to the union of the two existing journals under a new order of things, was inaugurated by Drs. Etheridge, Bridge and Hyde. These gentlemen, with others of their friends undertook the organization of a joint-stock company, to be entitled "The Chicago Medical Press Association," for the purpose of publishing a medical journal, and the establishment of a medical library and reading rooms for the benefit of the members of the Association.

The organization is now complete, the journals are united under the name of the CHICAGO MEDICAL JOURNAL AND EXAMINER, and the Medical Press Association is responsible for its editorial management.

This, we think, is sufficient guarantee for the scientific and practical character of its contents, and the purity of its ethics. It is also an assurance to the profession that the JOURNAL AND EXAMINER will be conducted in a spirit of true catholicity for the advancement of the science of medicine and the promotion of fraternity among physicians.

To place its success and permanency beyond question, we have only to add, that its business management and publication are entrusted to the extensive and widely known publishing house of W. B. Keen, Cooke & Co., of this city.

The editors invite all members of the profession to contribute original articles to the pages of the Journal and Examiner, and promise them, in return, that their communications will be appreciated and proper consideration bestowed upon them.

There are many men among us who possess the ability and qualifications to distinguish themselves, but whom reticence—from either diffidence or indifference—has withheld from their deserved positions. To them we appeal for aid and support in making a first-class periodical.

Our circulation for the remaining portion of the year is large, as our subscription list contains the names of all the subscribers of both the Chicago Medical Journal and the Medical Examiner, and we confidently anticipate that it will be more extensive next year; hence, our contributors by their communications will reach a great number of intelligent and appreciative readers.

Remembering that we have received our young protege from the hands of experienced and accomplished editors—men of extensive reputation and fame—we can but feel a sense of diffidence, and a distrust in our ability to maintain for the Journal and Examiner that high position which its two predecessors have attained through their agency. We promise, however, an earnest and industrious effort to succeed in that arduous undertaking.

Our facilities for obtaining foreign and domestic exchanges are such as to assure us that we shall soon be in receipt of the most valuable periodicals from the medical centres of the world. From them we hope to glean all new facts and discoveries upon their first appearance.

We expect to make the excerpt department of the

JOURNAL AND EXAMINER a very interesting and profitable one.

In our own city we are perfecting arrangements by which reports from the vario us Hospitals and Societies of Chicago will appear upon our pages in such a way as to make them a mirror of the practice of our industrious and accomplished corps of teachers and investigators.

We are already promised correspondence from many of the great cities of this country and Europe, and before long this also will be an important department.

Finally, we very respectfully salute our senior contemporaries in journalism, and bespeak for ourselves a fraternal reception among them, and we promise, in return, every effort to deserve well from them.

---

The *New York Medical Record*, of August 7, 1875, erroneously announces that "the CHICAGO MEDICAL EXAMINER, heretofore published by N. S. Davis, M.D., and F. Davis, M.D., is to be consolidated with the CHICAGO MEDICAL JOURNAL. After Aug. 1st it will be known as 'THE CHICAGO MEDICAL JOURNAL AND EXAMINER,' and will be edited by Drs. J. Adams Allen and Walter Hay."

We trust that the foregoing statement, which results from a total misapprehension of facts, will not be repeated or copied in other of our Exchanges. The Chicago Medical Press Association is a stock corporation, which includes various physicians of this city who represent, it is believed, all its different medical institutions and hospitals. *They* have succeeded in securing possession of *both* the medical journals heretofore published in Chicago, and have consolidated them under the name of the "CHICAGO MEDICAL JOURNAL AND EXAMINER." It will be seen that the editors of the new monthly, whose names appear in connection with this issue, are not those announced in the *Medical Record.* The former have been appointed to their editorial duties by the Board of Directors of the Chicago Medical Press Association.

